# Genome-Wide Characterization of the bZIP Gene Family in Winter Jujube Highlights *ZjbZIP7*’s Association with Late Fruit Development

**DOI:** 10.3390/genes17101217

**Published:** 2026-09-30

**Authors:** Shumin Li, Li Tian, Hongwei Chen, Huafeng Yue, Gaopu Zhu, Mengmeng Zhang

**Affiliations:** 1 School of Horticulture and Landscape Architecture, Henan Forestry Vocational College, Luoyang 471002, China; 2 College of Biology and Food Engineering, Anyang Institute of Technology, Anyang 455000, China; 3 Research Institute of Non-Timber Forestry, Chinese Academy of Forestry, Zhengzhou 450003, China

**Keywords:** *Ziziphus jujuba* Mill. cv. Dongzao, bZIP transcription factor, genome-wide analysis, fruit development, ethylene biosynthesis

## Abstract

**Background:** Basic leucine zipper (bZIP) transcription factors function widely in plant development, hormone signaling, and fruit ripening, but the bZIP family and its potential regulatory roles in winter jujube fruit development remain poorly characterized. **Methods:** a genome-wide analysis was performed using the gapless genome assembly of winter jujube (*Ziziphus jujuba* Mill. cv. Dongzao), including phylogenetic, chromosomal distribution, gene duplication, synteny, gene structure, conserved motif, and promoter cis-element analyses. Developmental expression patterns were investigated using RNA-seq and RT-qPCR. Weighted gene co-expression network analysis (WGCNA) and a yeast one-hybrid assay were further used to examine the co-expression relationships of ZjbZIP7 and its promoter-binding association with *ZjACO1*. **Results:** A total of 52 *ZjbZIP* genes were identified and classified into nine phylogenetic groups. These genes were unevenly distributed across 11 chromosomes, and 11 segmentally duplicated gene pairs were detected. Comparative synteny analysis identified 46 syntenic bZIP gene pairs between winter jujube and the sour jujube (*Z. jujuba* var. *spinosa*) and 42 between winter jujube and *Arabidopsis thaliana.* Promoter analysis identified 53 types of cis-acting elements associated with light, phytohormones, stress responses, and development; notably, abscisic acid-responsive elements were present in 47 of the 52 promoters. RNA-seq analysis across seven fruit developmental stages showed that 18 *ZjbZIP* genes accumulated preferentially during the late stages, with *ZjbZIP7* showing particularly pronounced transcript accumulation during the late developmental stages. RT-qPCR analysis of eight representative genes broadly confirmed their RNA-seq profiles. Weighted gene co-expression network analysis identified 38 genes tightly co-expressed with *ZjbZIP7*, including the ethylene biosynthesis gene *ZjACO1*. A yeast one-hybrid assay further demonstrated that ZjbZIP7 bound to the *ZjACO1* promoter in yeast. **Conclusions:** These findings enhance our understanding of the evolutionary relationships and developmental expression patterns of the *ZjbZIP* gene family as a whole and provide valuable candidate genes for elucidating fruit-ripening mechanisms that may be useful in improving fruit quality in winter jujube breeding.

## 1. Introduction

Basic leucine zipper (bZIP) proteins constitute a widespread family of transcription factors in eukaryotes and are characterized by a conserved bZIP domain. This domain consists of a basic region responsible for sequence-specific DNA binding and nuclear localization and an adjacent leucine zipper that mediates the formation of homodimers and heterodimers [1,2]. Different combinations of bZIP homodimers and heterodimers exhibit distinct DNA-binding preferences and consequently regulate different sets of target genes [3]. In plants, bZIP proteins mainly recognize ACGT-containing cis-elements, such as the A-box, C-box, G-box, and abscisic acid-responsive element (ABRE) [4,5,6]. bZIP proteins participate in seed maturation, organ development, carbon and nitrogen metabolism, secondary metabolism, hormone signaling, and responses to biotic and abiotic stresses [7]. In strawberry, the ABF/AREB-type bZIP transcription factor FvABF3 directly binds to ABREs in the *FvALKBH10B* promoter and activates its transcription, thereby promoting fruit ripening [8]. The capacity for dimerization and selective recognition of cis-regulatory elements enables bZIP proteins to integrate developmental, hormonal, and environmental signals.

The increasing availability of high-quality plant genomes has enabled the genome-wide identification and characterization of bZIP family members in numerous dicotyledonous and monocotyledonous species, including *A. thaliana* [9], *Oryza sativa* [10], *Zea mays* [11], *Triticum aestivum* [12], *Vitis vinifera* [13], *Cucumis sativus* [14], *Solanum lycopersicum* [15], *Malus* x *domestica* [16], *Musa acuminata* [17], *Citrullus lanatus* [18], *Populus trichocarpa* [19], *Allium sativum* [20], and *Lycium barbarum* [21]. These analyses have revealed substantial interspecific variation in bZIP family size, chromosomal distribution, duplication patterns, and subgroup composition. Despite this variation, the basic region and leucine zipper are broadly conserved across plant species, and genes assigned to the same phylogenetic group often share similar exon–intron structures and motif compositions. Cross-species comparisons can therefore assist with subgroup assignment and functional prediction, but species-specific analyses remain necessary to define the composition and to verify functions of the bZIP family within individual genomes.

Functional studies in several fruit species have shown that bZIP transcription factors contribute to fruit development and ripening. In apple, MdHY5 promotes anthocyanin accumulation by directly activating *MdMYB10* transcription and also influences nitrate assimilation, whereas MdbZIP44 promotes ABA-induced anthocyanin accumulation by enhancing the binding of MdMYB1 to the promoters of downstream anthocyanin biosynthetic genes [22,23]. In banana, MabZIP4 and MabZIP5 activate genes involved in aroma biosynthesis [24]. Phosphorylation of MabZIP93 by MaMPK2 enhances its transcriptional activation of genes involved in cell wall modification [25]. Conversely, MabZIP74 represses the ethylene biosynthesis genes *MaACO1* and *MaACO4*, and phosphorylation by MaMAPK11-3 weakens this repression [26]. MabZIP21 directly activates several ripening-associated genes, while its phosphorylation by MaMPK6-3 enhances its transcriptional activity and accelerates fruit ripening [27]. MaWRKY49 and MaWRKY111 also interact with MabZIP21 and cooperatively activate the transcription of *MaACS1* and *MaACO1*, further linking bZIP activity to ethylene biosynthesis [28]. These studies show that bZIP proteins participate in fruit coloration, aroma formation, cell wall remodeling, and ethylene production through direct promoter binding, interactions with other transcription factors, and MAPK-mediated phosphorylation. However, functional evidence remains limited to a small number of fruit species, and it remains unclear whether comparable regulatory modules operate in both climacteric and non-climacteric fruits.

Winter jujube (*Z. jujuba* Mill. cv. Dongzao) is an economically important fresh-market cultivar valued for its crisp texture, distinctive flavor, and high nutritional quality [29,30]. Winter jujube development and ripening are marked by changes in peel pigmentation and cell wall composition [31] as well as sugar and organic acid metabolism and flavor formation [30], while maturity at harvest affects postharvest quality and storability [32]. The first jujube genome and the subsequent gapless genome assembly of ‘Dongzao’ constitute valuable genomic resources for investigating the molecular regulation of these traits [33,34]. Although bZIP genes have previously been catalogued in another accession of jujube [35], the composition and chromosomal organization of the *ZjbZIP* family have not been re-evaluated using the gapless ‘Dongzao’ genome. Moreover, the developmental expression patterns and regulatory functions of these genes remain poorly characterized. In particular, it remains unclear which *ZjbZIP* genes are associated with fruit ripening and whether ZjbZIP proteins directly regulate ethylene biosynthetic genes.

Accordingly, this study aimed to systematically characterize the *bZIP* gene family in winter jujube using the gapless ‘Dongzao’ genome, including analyses of phylogenetic relationships, chromosomal distribution, duplication patterns, gene structures, conserved motifs, and promoter cis-regulatory elements. We further investigated the expression patterns of *ZjbZIP* genes across seven stages of fruit development to identify candidates associated with late fruit development. Finally, *ZjbZIP7* was selected for further analysis of its co-expression relationships and its promoter-binding association with ZjACO1 using a yeast one-hybrid assay. This study establishes a genomic and transcriptional framework for investigating bZIP-mediated regulation of winter jujube fruit maturation and supports future functional studies of genes relevant to fruit quality and postharvest performance.

## 2. Materials and Methods

### 2.1. Plant Materials

Fruit samples were collected in 2025 from healthy, 10-year-old winter jujube (*Z. jujuba* Mill. cv. Dongzao) trees grown in Anyang, Henan Province, China. Fruits were harvested at 40, 55, 70, 85, 100, 115, and 130 days after pollination (DAP) and designated as developmental stages S1–S7, respectively. At each developmental stage, fruits were collected from three independent trees, with each tree representing one biological replicate. For each replicate, 10 fruits of uniform size and developmental status and without mechanical damage or visible symptoms of pests or disease were collected from the same tree and pooled for subsequent analyses. Thus, three independent tree-based biological replicates were obtained at each developmental stage. Fruit tissues were immediately frozen in liquid nitrogen and stored at −80 °C until RNA extraction and subsequent analyses.

### 2.2. Identification of bZIP Gene Family Members in Winter Jujube

The predicted protein sequences and genome annotation files of winter jujube (*Z. jujuba* Mill. cv. Dongzao) were downloaded from the National Center for Biotechnology Information (NCBI) under assembly ASM3175591v1. The 78 annotated Arabidopsis bZIP protein sequences were retrieved from the Arabidopsis Information Resource (TAIR; https://www.arabidopsis.org/; accessed on 24 December 2025) and used as queries in BLASTP searches against the predicted winter jujube proteome using TBtools-II v2.154 [36]. An E-value cutoff of 1 × 10^−10^ was applied to retain candidate sequences. In parallel, the hidden Markov model profile corresponding to the conserved bZIP domain (Pfam accessions PF00170 and PF07716) was obtained from the Pfam database (http://pfam-legacy.xfam.org/; accessed on 24 December 2025) and used to search the winter jujube proteome with the HMMER web server [37] (https://www.ebi.ac.uk/Tools/hmmer/; accessed on 24 December 2025). Candidate proteins obtained from the BLASTP and HMMER searches were combined, and redundant sequences were removed. The remaining candidates were examined using NCBI Batch CD-Search (https://www.ncbi.nlm.nih.gov/Structure/cdd/wrpsb.cgi; accessed on 24 December 2025) and SMART [38] (https://smart.embl-heidelberg.de/; accessed on 24 December 2025). Proteins lacking a complete bZIP domain were excluded. The confirmed genes were designated ZjbZIP1 to ZjbZIP52 according to their physical positions on the chromosomes. The protein length, molecular weight (MW), theoretical isoelectric point (pI), instability index, aliphatic index, and grand average of hydropathicity (GRAVY) of each ZjbZIP protein were calculated using ExPASy ProtParam (https://web.expasy.org/protparam/; accessed on 26 December 2025). Proteins with instability indices below 40 were classified as stable, whereas those with values above 40 were classified as unstable. The predicted subcellular localization of each ZjbZIP protein was determined using WoLF PSORT (https://wolfpsort.hgc.jp/; accessed on 26 December 2025), with ‘plant’ selected as the organism type and the top-ranked localization recorded.

### 2.3. Phylogenetic, Chromosomal Distribution, Gene Duplication, and Synteny Analyses

The full-length amino acid sequences of the ZjbZIP proteins from winter jujube and Arabidopsis were aligned using the MUSCLE algorithm implemented in MEGA X v10.1.8. A neighbor-joining phylogenetic tree was constructed in MEGA X using the p-distance model. Sites containing gaps or missing data were treated using the partial-deletion option, and branch support was evaluated using 1000 bootstrap replicates. The resulting phylogenetic tree was annotated and visualized using Evolview v2 (https://evolgenius.info/evolview-v2/#login; accessed on 26 December 2025).

The chromosomal coordinates of the ZjbZIP genes were extracted from the winter jujube genome annotation file and visualized using TBtools v2.154. Gene duplication events and intragenomic collinear relationships among the ZjbZIP genes were identified using MCScanX [39]. Comparative synteny relationships between jujube and *Arabidopsis* and between winter jujube and Chinese sour jujube (*Z. jujuba* Mill. var. *spinosa*) were analyzed using MCScanX and visualized using the Dual Synteny Plot module in TBtools v2.154.

### 2.4. Conserved Motif, Gene Structure, and Cis-Acting Element Analyses of ZjbZIP Family Members

Conserved motifs in the amino acid sequences of ZjbZIP family members were analyzed using the MEME online tool (http://memesuite.org/tools/meme; accessed on 28 December 2025). Subsequently, the exon–intron structures of the corresponding *ZjbZIP* genes were analyzed using GSDS 2.0 (http://gsds.gaolab.org; accessed on 28 December 2025) based on the winter jujube GFF3 annotation file. The motif distributions and gene structures were subsequently integrated and visualized using TBtools v2.142.

The 2000 bp genomic sequences upstream of the annotated transcription start site (TSS) of each ZjbZIP gene were extracted from the winter jujube genome using TBtools and used as putative promoter regions. The extracted sequences were submitted to PlantCARE (http://bioinformatics.psb.ugent.be/webtools/plantcare/html; accessed on 28 December 2025) to predict putative cis-acting regulatory elements. The predicted results were visualized using TBtools v2.142.

### 2.5. Expression Analysis of ZjbZIP Genes During Winter Jujube Fruit Development

RNA-seq data from winter jujube fruit collected at seven developmental stages (S1–S7), comprising three biological replicates per stage and 21 libraries in total, were obtained from our previously generated transcriptomic dataset deposited in the NCBI Sequence Read Archive under BioProject accession PRJNA1173509 [40]. Detailed procedures for RNA extraction, library construction, sequencing, read quality control, alignment, reference genome annotation, and transcript quantification were described in the original study [40]. In the present study, the previously generated expression matrix was reused to investigate the temporal expression patterns of the ZjbZIP genes. FPKM values for individual ZjbZIP genes were extracted from the expression matrix, and the mean FPKM value at each developmental stage was calculated from the three biological replicates. For each gene, FPKM values across the seven developmental stages were standardized by row using Z-score transformation. The standardized expression values were visualized as a heatmap using TBtools v2.142. Genes showing preferential transcript accumulation at S6 and/or S7 relative to the earlier developmental stages were classified as late-stage-associated ZjbZIP genes.

### 2.6. RNA Extraction and RT-qPCR Validation

Total RNA was extracted from winter jujube fruit samples using a plant total RNA extraction kit (Vazyme, Nanjing, China). RNA concentration and purity were assessed using a NanoDrop 2000 spectrophotometer (Thermo Fisher Scientific, Waltham, MA, USA), and high-quality RNA was reverse-transcribed using HiScript III RT SuperMix for qPCR (+gDNA wiper) (Vazyme, Nanjing, China). Gene-specific primers for the selected *ZjbZIP* genes were designed using NCBI Primer-BLAST (Primer3 v2.5.0, accessed on 13 April 2026) (Table S1). RT-qPCR was performed using ChamQ Universal SYBR qPCR Master Mix (Vazyme, Nanjing, China) on an ABI 7500 Fast Real-Time PCR System (Applied Biosystems, Foster City, CA, USA). Three independent biological replicates were analyzed for each developmental stage, and each biological replicate was measured using three technical replicates. Amplification specificity was confirmed by melting-curve analysis, and primer amplification efficiencies were determined using standard curves generated from serially diluted cDNA. The housekeeping gene *ZjActin* (GenBank accession KT381859) was used as the internal reference for normalization, and relative expression levels were calculated by the 2^−ΔΔCt^ method [41].

### 2.7. Weighted Gene Co-Expression Network Analysis

Weighted gene co-expression network analysis was performed using the WGCNA package in R based on 21 RNA-seq libraries from seven fruit developmental stages. FPKM values were transformed using log_2_(FPKM + 1), and genes with negligible expression were removed. The soft-thresholding power was determined using the pickSoftThreshold function. A soft-thresholding power of β = 10 was selected based on the scale-free topology criterion. Co-expression modules were identified using dynamic tree cutting with a minimum module size of 30 and merged at mergeCutHeight = 0.25; module eigengenes and module membership (kME) were subsequently calculated. Module eigengenes were calculated to summarize the overall expression pattern of each module, and module membership (kME), defined as the correlation between the expression profile of an individual gene and the corresponding module eigengene, was calculated for genes within each module. The module containing *ZjbZIP7* was selected for further analysis, and a connection weight greater than 0.6 was used as a visualization cutoff to retain relatively stronger associations with *ZjbZIP7* for visualization in Cytoscape v3.7.1.

### 2.8. Yeast One-Hybrid Assay

The *ZjACO1* promoter fragment containing predicted bZIP-binding elements was amplified from winter jujube genomic DNA and inserted into pAbAi to generate pAbAi-Pro*ZjACO1*. The linearized construct was integrated into the Y1HGold genome. Background AbA resistance was evaluated on SD/−Ura medium containing different concentrations of aureobasidin A (AbA), and 200 ng mL^−1^ AbA was selected for interaction screening. The full-length coding sequence of *ZjbZIP7* was inserted into pGADT7 and transformed into the bait strain. Yeast cultures were adjusted to the same OD_600_ and subjected to 10-fold serial dilutions from 10^−1^ to 10^−4^. Aliquots were spotted onto SD/−Leu and SD/−Leu/AbA media and incubated at 30 °C for 3–5 days. The AD-Rec-53/p53-Promoter and AD-Empty/ProZjACO1 combinations were used as the positive and negative controls, respectively. Yeast growth on SD/−Leu/AbA medium was used to evaluate the binding of ZjbZIP7 to the *ZjACO1* promoter.

## 3. Results

### 3.1. Genome-Wide Identification and Characterization of bZIP Genes in Winter Jujube

To identify candidate ZjbZIP proteins, the Arabidopsis bZIP protein sequences were used as queries in BLAST searches against the predicted proteome of winter jujube (*Z. jujuba* Mill. cv. Dongzao). The resulting candidate sequences were examined using SMART and NCBI Batch CD-Search, and proteins lacking a complete bZIP domain were excluded. A total of 52 *ZjbZIP* genes were identified and named *ZjbZIP1* to *ZjbZIP52* according to their chromosomal positions (Table S2). The ZjbZIP proteins ranged from 127 amino acids in ZjbZIP49 to 749 amino acids in ZjbZIP42, with predicted molecular weights ranging from 14.67 kDa to 82.15 kDa. The predicted isoelectric points (pI) varied from 4.61 for ZjbZIP7 to 9.96 for ZjbZIP49. The instability indices ranged from 34.20 for ZjbZIP1 to 78.92 for ZjbZIP8. The instability indices of ZjbZIP1, ZjbZIP9, ZjbZIP34, and ZjbZIP52 were below 40, and these four proteins were therefore classified as stable. In contrast, the remaining 48 proteins were predicted to be unstable. The aliphatic indices ranged from 48.21 for ZjbZIP8 to 99.77 for ZjbZIP18. All ZjbZIP proteins had negative values for GRand AVerage of hYdropathicity (GRAVY), ranging from −1.084 for ZjbZIP14 to −0.312 for ZjbZIP18. Subcellular localization prediction further indicated that 49 of the 52 ZjbZIP proteins would localize to the nucleus, whereas ZjbZIP18, ZjbZIP27, and ZjbZIP42 were predicted to localize to the cytosol, peroxisome, and endoplasmic reticulum, respectively.

### 3.2. Phylogenetic Relationships, Chromosomal Distribution, Gene Duplication, and Synteny of ZjbZIP Genes

**Figure 1:**
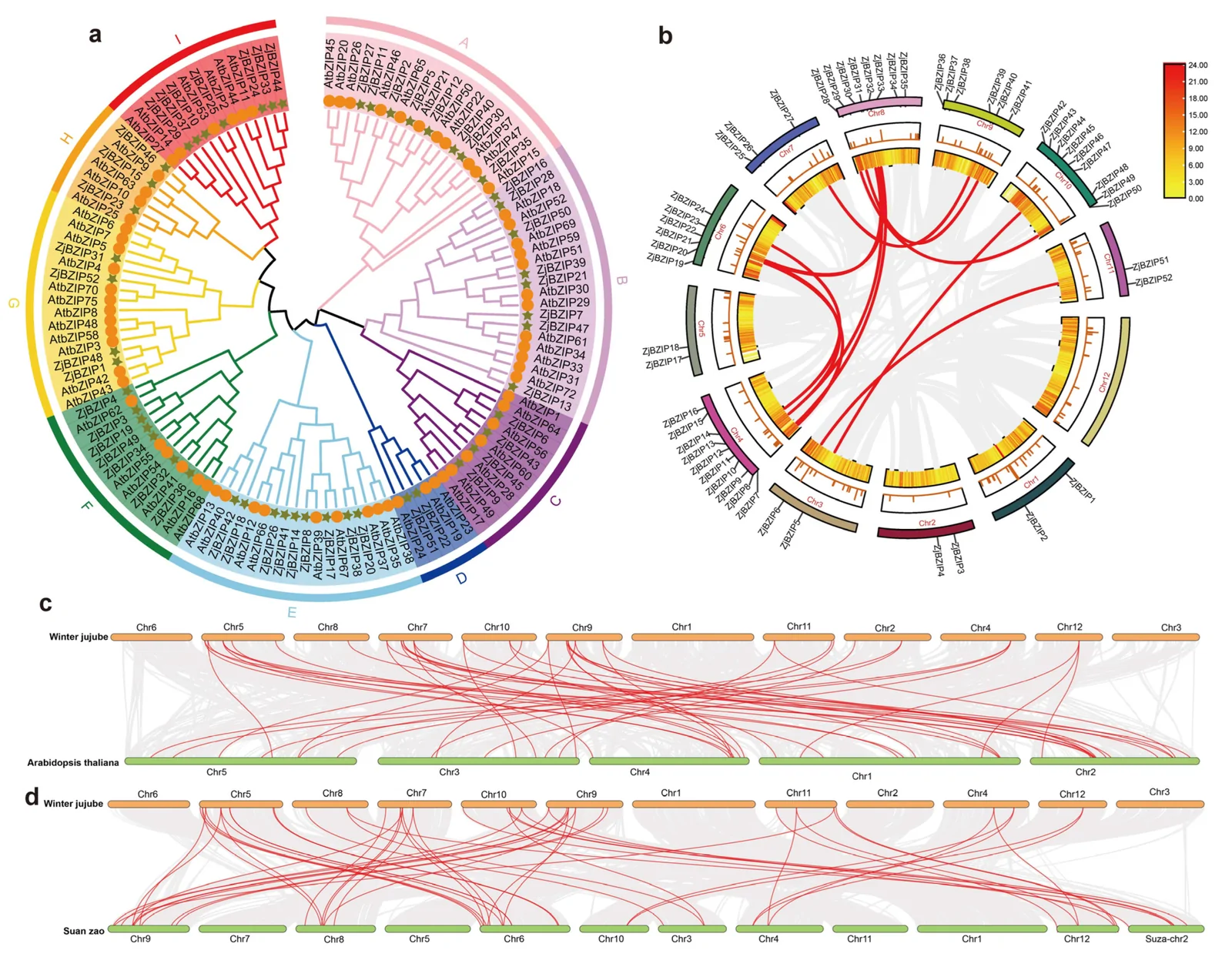
Phylogenetic relationships, chromosomal distribution, and synteny analyses of *ZjbZIP* genes in winter jujube. (**a**) Neighbor-joining phylogenetic tree constructed using ZjbZIP and AtbZIP protein sequences from winter jujube (*Z. jujuba* Mill. cv. Dongzao) and *Arabidopsis*, respectively. Green stars indicate ZjbZIP proteins, whereas orange circles indicate AtbZIP proteins. Different branch and outer-ring colors represent the nine phylogenetic groups (A–I). (**b**) Chromosomal distribution and intraspecific synteny of ZjbZIP genes in winter jujube. (**c**,**d**) Comparative synteny maps between winter jujube and *A. thaliana* and between winter jujube and Chinese sour jujube (*Z. jujuba* Mill. var. *spinosa*), respectively. Red lines indicate syntenic relationships involving *ZjbZIP* genes across the compared genomes.

The evolutionary relationships of the ZjbZIP family were examined by constructing a neighbor-joining phylogenetic tree using ZjbZIP and AtbZIP protein sequences (Figure 1a). Based on the topology of the phylogenetic tree, the bZIP proteins from winter jujube and *Arabidopsis* were classified into nine groups, designated A–I. Groups A–I contained 8, 8, 4, 2, 9, 7, 4, 3, and 7 ZjbZIP members, respectively. ZjbZIP and AtbZIP proteins were present in all nine groups, and several ZjbZIP proteins clustered with AtbZIP proteins within the same subclades. Chromosomal mapping showed that the 52 *ZjbZIP* genes were unevenly distributed across the jujube chromosomes (Figure 1b). Chr04 contained the largest number of *ZjbZIP* genes, with 10 members, followed by Chr10, Chr08, and Chr06, which contained nine, eight, and six genes, respectively. In contrast, Chr01, Chr02, Chr03, Chr05, and Chr11 each contained only two *ZjbZIP* genes. Intraspecific synteny analysis identified 11 segmentally duplicated *ZjbZIP* gene pairs, whose constituent genes were distributed across Chr03, Chr04, Chr06, Chr07, Chr08, Chr09, Chr10, and Chr11 (Figure 1b). Comparative synteny analysis identified 46 syntenic bZIP gene pairs between winter jujube and sour jujube and 42 pairs between winter jujube and *Arabidopsis* (Figure 1c,d). The syntenic genes were distributed across multiple chromosomes in the three genomes. Several *ZjbZIP* genes showed syntenic relationships with more than one homologous locus in either the sour jujube or Arabidopsis genome. These cross-species syntenic relationships indicated conservation of the bZIP gene family among winter jujube, Chinese sour jujube, and *Arabidopsis*.

### 3.3. Conserved Motif Composition and Gene Structure of ZjbZIP Family Members

**Figure 2:**
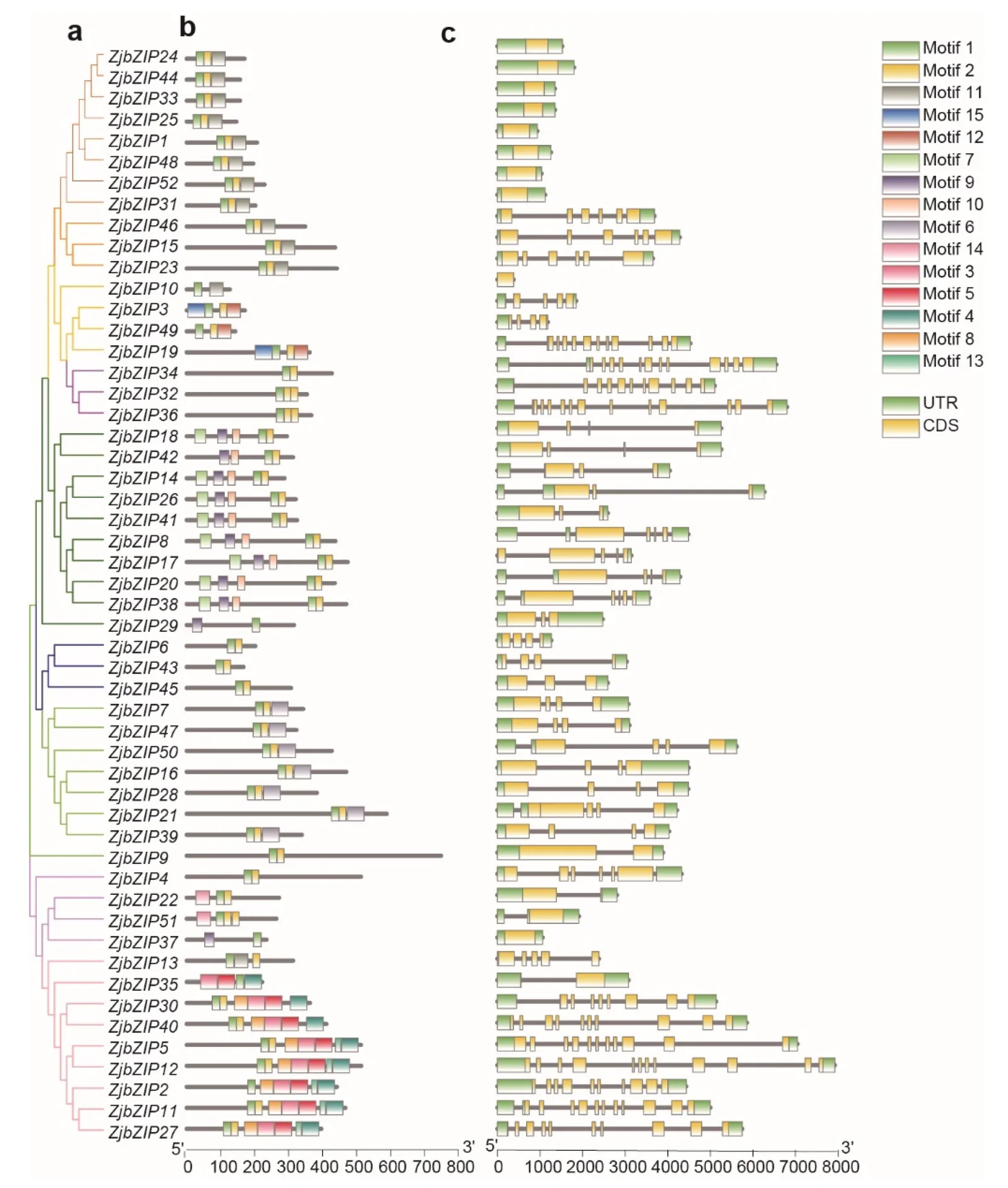
Phylogenetic relationships, conserved motif composition, and exon–intron structures of ZjbZIP family members. (**a**) Neighbor-joining phylogenetic tree of the 52 ZjbZIP proteins. (**b**) Distribution of motifs in the ZjbZIP proteins. Boxes of different colors represent individual motifs, and their positions indicate the corresponding locations within the protein sequences. (**c**) Exon–intron structures of the *ZjbZIP* genes. Green boxes, yellow boxes, and gray lines represent untranslated regions (UTRs), coding sequences (CDSs), and introns, respectively.

To characterize the structural features of the *ZjbZIP* family, the phylogenetic relationships, conserved motif compositions, and exon–intron structures of all 52 *ZjbZIP* family members were examined (Figure 2). The phylogenetic tree of the 52 ZjbZIP proteins contained the same nine groups identified in the ZjbZIP and AtbZIP phylogeny presented in Figure 1a (Figure 2a). Conserved motif analysis revealed that ZjbZIP proteins belonging to the same phylogenetic group generally shared similar motif composition and arrangement (Figure 2b). Motif 1 was detected in all 52 ZjbZIP proteins, whereas motif 2 was absent from ZjbZIP10, ZjbZIP29, ZjbZIP35, and ZjbZIP37. Eight proteins, ZjbZIP4, ZjbZIP6, ZjbZIP9, ZjbZIP10, ZjbZIP29, ZjbZIP37, ZjbZIP43, and ZjbZIP45, each contained only two of the 15 conserved motifs, which was the lowest motif number observed among the ZjbZIP proteins. Gene structure analysis further showed that the 52 *ZjbZIP* genes contained between 3 and 13 exons (Figure 2c). Genes assigned to the same phylogenetic group generally exhibited similar exon–intron arrangements and intron numbers.

### 3.4. Cis-Acting Regulatory Elements in the Promoters of ZjbZIP Genes

**Figure 3:**
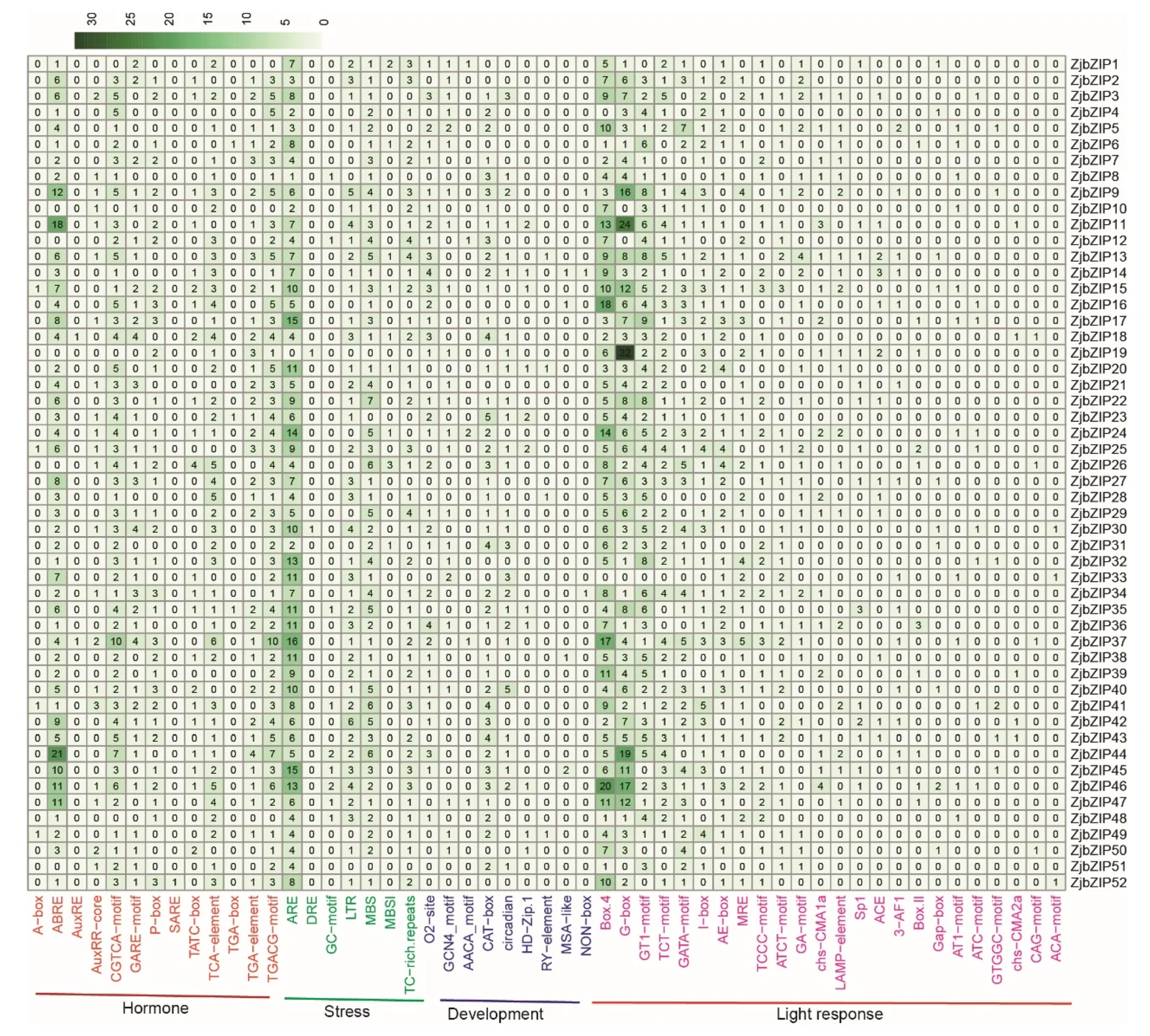
Distribution of cis-acting regulatory elements in the promoter regions of the 52 *ZjbZIP* genes. Rows represent the *ZjbZIP* genes, and columns represent 53 cis-acting element types grouped into hormone-responsive, stress-responsive, development-related, and light-responsive categories. Numbers within the cells indicate the numbers of occurrences of individual elements in the corresponding promoters. The green color gradient represents element abundance, with darker shades indicating greater numbers of occurrences.

To investigate the potential transcriptional regulation of *ZjbZIP* genes, putative cis-acting regulatory elements in their promoter regions were identified using PlantCARE (Figure 3). Across the 52 promoters, 53 distinct cis-acting element types were identified and assigned to four categories: light-responsive elements (24 types), hormone-responsive elements (13 types), development-related elements (9 types), and stress-responsive elements (7 types). The light-responsive category contained the largest number of element types. Within this category, Box 4 and G-box were the most frequently detected elements, occurring 341 and 303 times, respectively.

The hormone-responsive elements were associated with responses to abscisic acid (ABA), methyl jasmonate (MeJA), auxin, gibberellin, and salicylic acid (SA). ABRE was the most frequently detected hormone-responsive element, with 233 occurrences distributed across 47 of the 52 *ZjbZIP* promoters (90.4%). The MeJA-responsive TGACG-motif and CGTCA-motif were also widely distributed, occurring 153 and 152 times, respectively. The promoter of *ZjbZIP44* contained the highest number of ABREs (21) and seven TGACG-motifs. Auxin-responsive elements, including AuxRE, AuxRR-core, and TGA-element, occurred a total of 86 times, whereas the gibberellin-responsive GARE-motif, P-box, and TATC-box occurred a total of 144 times.

Among the stress-responsive elements, antioxidant responsive elements (AREs) were the most frequently detected, with 371 occurrences, followed by the drought-responsive MBS (132) and low-temperature-responsive LTR (85). Among the development-related elements, CAT-box, O2-site, and circadian elements occurred 84, 58, and 41 times, respectively. The number and composition of cis-acting regulatory elements varied among the 52 *ZjbZIP* promoters.

### 3.5. Expression Patterns of ZjbZIP Genes Across Seven Stages of Fruit Development

**Figure 4:**
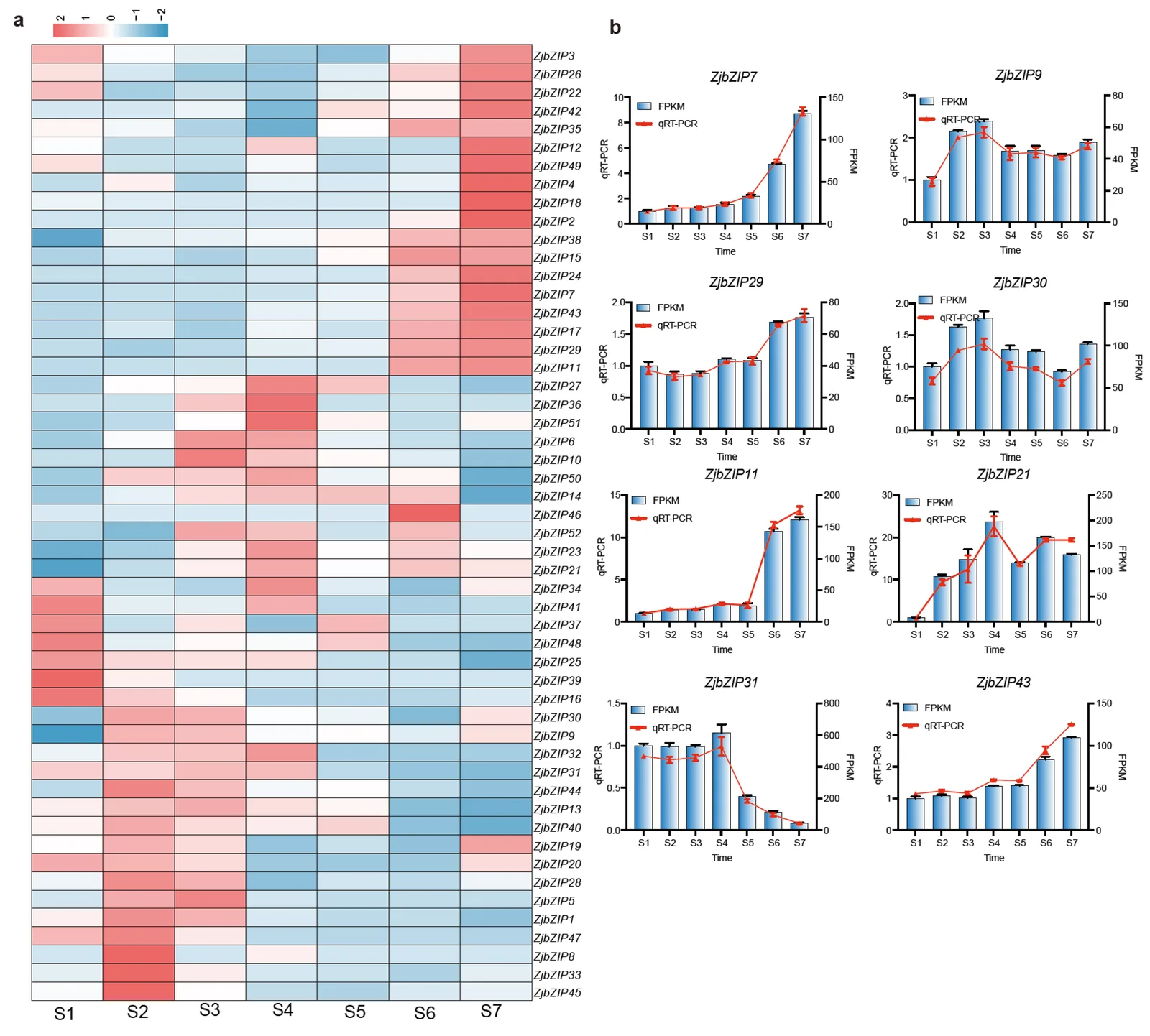
Expression profiles of *ZjbZIP* genes during seven stages of winter jujube fruit development. (**a**) Heatmap showing the RNA-seq expression profiles of 52 *ZjbZIP* genes from fruit stage name (S1) to full maturity (S7). The color scale represents standardized expression values, with red and blue indicating relatively high and low expression levels, respectively. (**b**) Comparison of the RNA-seq and RT-qPCR expression profiles of eight selected *ZjbZIP* genes. Blue bars represent RNA-seq FPKM values shown on the right *y*-axis, whereas red lines represent relative RT-qPCR expression levels shown on the left *y*-axis. Error bars represent the standard deviation (SD) of three independent biological replicates.

To characterize the transcriptional dynamics of the *ZjbZIP* family during fruit development, the expression profiles of 52 *ZjbZIP* genes were analyzed at seven developmental stages (S1 to S7) using RNA-seq data. The *ZjbZIP* genes exhibited diverse temporal expression patterns (Figure 4a). Based on their standardized temporal expression profiles, 18 *ZjbZIP* genes, including *ZjbZIP7*, *ZjbZIP11*, and *ZjbZIP43*, showed relatively high transcript levels during the late developmental stages, particularly at S6 and S7. In contrast, *ZjbZIP9* and *ZjbZIP30* showed relatively high expression at S2 and S3, *ZjbZIP21* reached its highest expression level at S4, and the expression of *ZjbZIP31* decreased markedly after S4. To validate the RNA-seq expression patterns, eight representative genes, *ZjbZIP7*, *ZjbZIP9*, *ZjbZIP11, ZjbZIP21*, *ZjbZIP29*, *ZjbZIP30*, *ZjbZIP31*, and *ZjbZIP43*, were selected for RT-qPCR analysis. The temporal expression trends determined by RT-qPCR were broadly consistent with the corresponding RNA-seq profiles (Figure 4b). Among these genes, the expression of *ZjbZIP7* increased progressively from S4 to S7 and reached its highest level at S7. Based on its marked transcript accumulation during the late developmental stages, *ZjbZIP7* was selected for subsequent characterization.

### 3.6. Co-Expression Network Analysis and Molecular Characterization of ZjbZIP7 During Fruit Ripening

**Figure 5:**
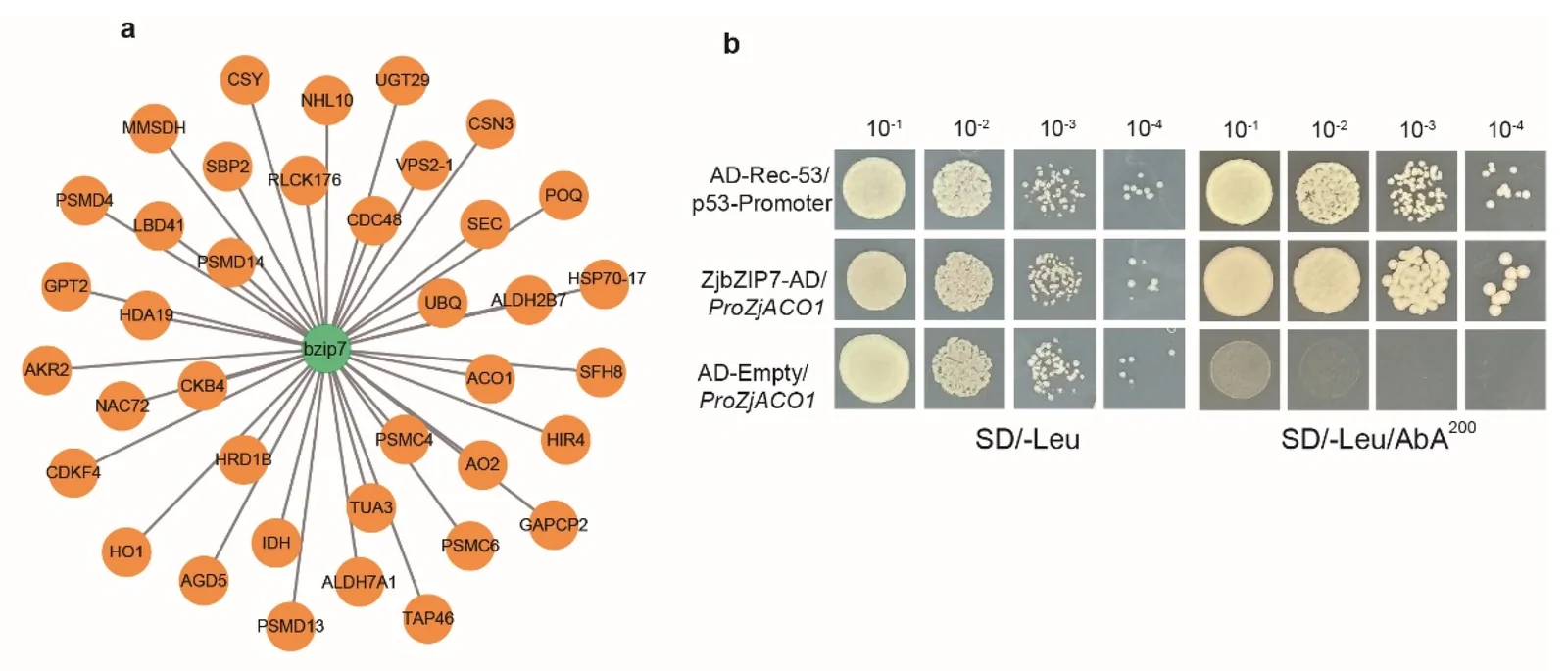
Co-expression network and promoter-binding analysis of ZjbZIP7. (**a**) WGCNA-derived co-expression network showing associations with *ZjbZIP7*. The central green node represents *ZjbZIP7*, the orange nodes represent 38 co-expressed genes with connection weights greater than 0.6, and the lines indicate co-expression relationships. (**b**) Yeast one-hybrid assay examining the binding of ZjbZIP7 to the *ZjACO1* promoter. Yeast cultures were serially diluted from 10^−1^ to 10^−4^ and grown on SD/−Leu medium or SD/−Leu medium supplemented with 200 ng mL^−1^ AbA. The AD-Rec-53/p53-Promoter and AD-Empty/ProZjACO1 combinations were used as the positive and negative controls, respectively. AbA, aureobasidin A.

To explore genes showing expression patterns associated with *ZjbZIP7* during fruit ripening, weighted gene co-expression network analysis (WGCNA) was performed using transcriptomic profiles from seven stages of fruit development (Figure S1). Using a soft-thresholding power of β = 10, a minimum module size of 30, and a module-merging threshold of 0.25, co-expression modules were constructed, and the module containing *ZjbZIP7* was selected for further analysis. Within this module, 38 genes were connected with *ZjbZIP7*, with connection weights greater than 0.6 (Figure 5a). These genes included *ZjACO1*, *ZjNAC72*, *ZjHSP70-17*, *ZjAO2*, and *ZjALDH2B7*. These co-expression relationships suggested that ZjbZIP7 may be associated with fruit ripening.

Among the genes associated with ZjbZIP7 in the co-expression network, promoter sequence analysis identified several putative bZIP-binding cis-elements in the *ZjACO1* promoter. A yeast one-hybrid (Y1H) assay was subsequently performed to examine whether ZjbZIP7 could bind to this promoter. All yeast transformants grew normally on SD/−Leu medium, confirming successful transformation. Yeast carrying the ZjbZIP7-AD/*ProZjACO1* combination grew on SD/−Leu medium supplemented with 200 ng mL^−1^ aureobasidin A (AbA) at all tested serial dilutions. Similar growth was observed for the positive control carrying AD-Rec-53/p53-Promoter. In contrast, the negative control carrying AD-Empty/*ProZjACO1* failed to grow under the same selective conditions (Figure 5b). These results showed that ZjbZIP7 could bind to the *ZjACO1* promoter in yeast, supporting *ZjACO1* as a candidate downstream target of ZjbZIP7 during fruit ripening.

## 4. Discussion

The bZIP gene family is widespread across the Plant Kingdom. However, the size of the bZIP gene family differs markedly among plant species, as shown by the identification of 52 bZIP genes in safflower (*Carthamus tinctorius*) [42], 55 in litchi (*Litchi chinensis*) [43], 66 in licorice (*Glycyrrhiza uralensis*) [44], and 160 in soybean (*Glycine max*) [45]. In the present study, 52 *ZjbZIP* genes were identified from the gapless ‘Dongzao’ genome. These differences in family size between species may reflect lineage-specific patterns of gene duplication, retention, and loss, as well as differences in ploidy [46]. Genome assembly and annotation quality can also influence estimates of gene family size. For example, genome-wide reannotation of bZIP genes in nine Solanaceae species identified 107 genes that were absent from previous annotations [47]. The gapless ‘Dongzao’ genome consequently provides an improved genomic framework for defining the composition and chromosomal organization of the *ZjbZIP* family [34]. The ZjbZIP proteins also displayed considerable variation in length, molecular weight, theoretical isoelectric point, and instability index. All ZjbZIP proteins were predicted to be hydrophilic and most to localize to the nucleus, consistent with their expected roles as transcription factors. Comparable variation in protein size and isoelectric point has been reported for bZIP proteins in safflower, litchi, and soybean [42,43,45], and most bZIP proteins are predicted to localize to the nucleus, including those in *Stevia rebaudiana* [48]. While the conserved bZIP domain is embedded within proteins with diverse sequence compositions, the variation in predicted physicochemical properties alone does not demonstrate evolutionary or functional divergence, and the predicted protein stability and subcellular localization of these proteins require experimental verification.

The *ZjbZIP* genes were unevenly distributed across 11 of the 12 winter jujube chromosomes, with the largest numbers in Chr04, Chr10, Chr08, and Chr06. Nonuniform chromosomal distributions of bZIP genes have also been reported in litchi, *Isatis indigotica*, *Stevia rebaudiana*, and soybean [43,45,48,49]. In winter jujube, 11 segmentally duplicated *ZjbZIP* gene pairs were identified, most of which were located on different chromosomes (Figure 1). This result indicates that segmental duplication contributed to the retention and evolution of the *ZjbZIP* family. Segmental duplication has also contributed to the evolution of bZIP families in other plant species. For example, 39 segmental duplication events were detected in litchi [43], 16 segmentally duplicated gene pairs were identified in *Isatis indigotica* [49], and 27 segmentally duplicated pairs were detected in *Stevia rebaudiana* [48]. In contrast, tandem duplication was reported as the predominant duplication mechanism in the licorice bZIP family [44], whereas both segmental and tandem duplications contributed to the evolution of the soybean bZIP family [45]. These differences indicate that the relative contributions of individual duplication mechanisms vary among plant lineages. Comparative genomic studies indicate that duplication mechanism and age can influence duplicate-gene retention, selective constraints, and expression divergence [46,50,51]. Following duplication, gene copies may be lost, retain overlapping functions, or undergo subfunctionalization or neofunctionalization [46]. Because synonymous and nonsynonymous substitution rates were not analyzed in the present study, the selective pressures acting on duplicated *ZjbZIP* gene pairs remain undetermined. The current evidence therefore supports a contribution of segmental duplication to the evolution and maintenance of the *ZjbZIP* family but does not establish that the duplicated genes have acquired new or specialized functions.

Promoter composition and developmental expression patterns provide complementary clues for predicting the potential functions of *ZjbZIP* genes. The promoters of the 52 *ZjbZIP* genes contained 53 types of predicted cis-acting elements associated with light, hormones, stress responses, and plant growth and development (Figure 3). Light-responsive elements were the most diverse, with Box 4 and G-box elements occurring frequently. Plant bZIP proteins commonly recognize ACGT-containing sequences, including the G-box, and the bZIP factor HY5 directly regulates G-box-containing promoters in light-signaling networks [52]. The abundance of G-boxes in *ZjbZIP* promoters therefore raises the possibility that some family members are transcriptionally regulated by other bZIP proteins, including light-responsive bZIP factors. Hormone-responsive elements were also widely distributed. ABREs occurred in 47 of the 52 promoters, while MeJA-, auxin-, gibberellin-, and salicylic acid-responsive elements were present in different subsets of genes. The broad distribution of ABREs is consistent with the central role of ABF/AREB-type bZIP proteins in ABA-responsive transcription [53]. In citrus, CsbZIP44 forms a transcriptional regulatory module with CsHB5 and directly activates carotenoid-related genes, thereby promoting ABA-mediated carotenoid accumulation and fruit coloration [54]. Citrus and deciduous jujube are both non-climacteric fruit, and this finding provides evidence that bZIP proteins can connect ABA signaling with ripening-associated metabolic changes in such fruit. However, the presence and frequency of predicted cis-elements do not establish promoter responsiveness; hormone treatments and promoter-activity assays are required to test these hypotheses.

The RNA-seq analysis revealed distinct temporal expression patterns among the *ZjbZIP* genes across the seven stages of winter jujube fruit development. Some genes were preferentially expressed during the early or intermediate stages, whereas 18 genes, including *ZjbZIP7*, *ZjbZIP11*, and *ZjbZIP43*, showed marked transcript accumulation at S6 and S7 (Figure 4). The early and intermediate expression patterns may reflect roles in developmental processes that precede ripening, whereas the genes with increased expression at S6 and S7 are candidates associated with maturation-related changes. Comparable stage-specific expression patterns have been observed in other fruit species. In litchi, *LITCHI018843.m1* showed preferential expression during late fruit development [43]. In Chinese pear, *PbZIP49* and *PbZIP59* were highly expressed during late development and senescence, whereas *PbZIP33*, *PbZIP6*, and *PbZIP9* declined as fruit development progressed [55]. Functional studies have further shown that some bZIP proteins regulate ripening-associated metabolism. In another pear species (*Pyrus ussuriensis* Maxim.), *PubZIP914* expression increases at fruit maturity, and the encoded protein directly activates *PuLOX3.1*, thereby promoting the production of fatty acid-derived volatiles [56]. In citrus, CsbZIP44 directly activates several carotenoid-metabolism genes and participates in ABA-mediated fruit coloration [54]. These observations support the prioritization of late-expressed *ZjbZIP* genes for analysis of their roles in regulating peel coloration, aroma formation, and hormone responses during non-climacteric jujube ripening. Nevertheless, temporal expression alone does not establish regulatory function, and further experimental validation is required.

The pronounced increase in *ZjbZIP7* transcript abundance during S6 and S7 suggests that this gene may be involved in the late stages of winter jujube fruit development. WGCNA further identified 38 genes strongly co-expressed with *ZjbZIP7*, including *ZjACO1*, *ZjNAC72*, *ZjHSP70-17*, *ZjAO2*, and *ZjALDH2B7*. However, co-expression indicates association and does not establish direct regulation or causality. Among these genes, *ZjACO1* is particularly relevant, because ACC oxidase catalyzes the conversion of 1-aminocyclopropane-1-carboxylic acid (ACC) to ethylene in the final enzymatic step of ethylene biosynthesis [57]. Previous studies have shown that bZIP proteins can regulate ethylene-biosynthesis genes through different mechanisms. In banana, MabZIP74 directly represses the transcription of *MaACO1* and *MaACO4*, whereas phosphorylation of MabZIP74 by MaMAPK11-3 attenuates this repression [26]. In contrast, MabZIP21 activates *MaACO1* and other ripening-associated genes, and phosphorylation of MabZIP21 by MaMPK6-3 enhances its transcriptional activity and promotes banana fruit ripening [27]. MaWRKY49 and MaWRKY111 also interact with MabZIP21 and synergistically enhance the transcription of *MaACS1* and *MaACO1* [28]. In papaya, CpbZIP52 directly binds to and activates the *CpACS2* promoter, and transient overexpression and silencing of *CpbZIP52* accelerate and delay fruit ripening, respectively [58]. Conversely, the pear bZIP protein PuHY5 binds to the *PuACS1* promoter and represses its transcription under blue light, thereby reducing ethylene production [59]. These findings indicate that bZIP proteins can positively or negatively regulate ethylene biosynthesis depending on the species and regulatory context. In the present study, the Y1H assay showed that ZjbZIP7 could bind to the tested *ZjACO1* promoter fragment in yeast, providing evidence for a potential promoter-binding association between ZjbZIP7 and *ZjACO1*. However, whether this interaction occurs in planta and whether ZjbZIP7 activates or represses *ZjACO1* transcription remain unresolved. Further transcriptional reporter assays in planta binding analyses, genetic manipulation of *ZjbZIP7*, and measurements of *ZjACO1* expression and ethylene production will be required to determine the regulatory direction and physiological significance of this interaction.

## 5. Conclusions

In this study, 52 *ZjbZIP* genes were identified from the gapless genome assembly of winter jujube and classified into nine phylogenetic groups (A–I). These genes were unevenly distributed across 11 of the 12 chromosomes, and eleven segmentally duplicated *ZjbZIP* gene pairs were identified. Between this cultivar and sour jujube, 46 syntenic *bZIP* gene pairs were identified. The *ZjbZIP* genes contained 3–13 exons, with members of the same phylogenetic group generally exhibiting similar exon–intron structures. Promoter analysis identified 53 types of cis-acting elements associated with light, phytohormones, stress responses, and development. Among them, ABREs occurred 233 times and were present in 47 of the 52 *ZjbZIP* promoters. One sequence motif, Motif 1, was conserved in all 52 ZjbZIP proteins. Expression profiling across seven fruit developmental stages showed that 18 genes, including *ZjbZIP7*, *ZjbZIP11*, and *ZjbZIP43*, exhibited preferential transcript accumulation at S6 and S7. Notably, *ZjbZIP7* expression increased progressively from S4 to S7 and reached its highest level at S7. WGCNA identified 38 genes associated with *ZjbZIP7* at connection weights greater than 0.6, including the ethylene biosynthesis gene *ZjACO1*. Furthermore, the yeast one-hybrid assay showed that ZjbZIP7 could bind to the tested *ZjACO1* promoter fragment in yeast. These findings provide genomic and expression resources for the *ZjbZIP* family and provide a basis for future investigation of the ZjbZIP7–*ZjACO1* relationship in planta.

## Data Availability

The original contributions presented in the study are included in the article/Supplementary Materials; further inquiries can be directed to the corresponding authors.

## Supplementary Materials

The following supporting information can be downloaded at: https://www.mdpi.com/article/10.3390/genes17101217/s1, Table S1: Primer sequences used for RT-qPCR analysis; Table S2: Physicochemical properties and predicted subcellular localizations of the 52 ZjbZIP proteins; Figure S1: Weighted gene co-expression network analysis of transcriptomic data from seven fruit developmental stages.
