# Supplementary material for: Genome-Wide Characterization of the bZIP Gene Family in Winter Jujube Highlights ZjbZIP7’s Association with Late Fruit Development
Source: Genes (Basel). 2026 Sep 30;17(10):1217. doi: 10.3390/genes17101217 (PMC13704098; doi:10.3390/genes17101217)
Supplement: Supplementary file 1 [file genes-17-01217-s001.zip › Figure S1.pdf]

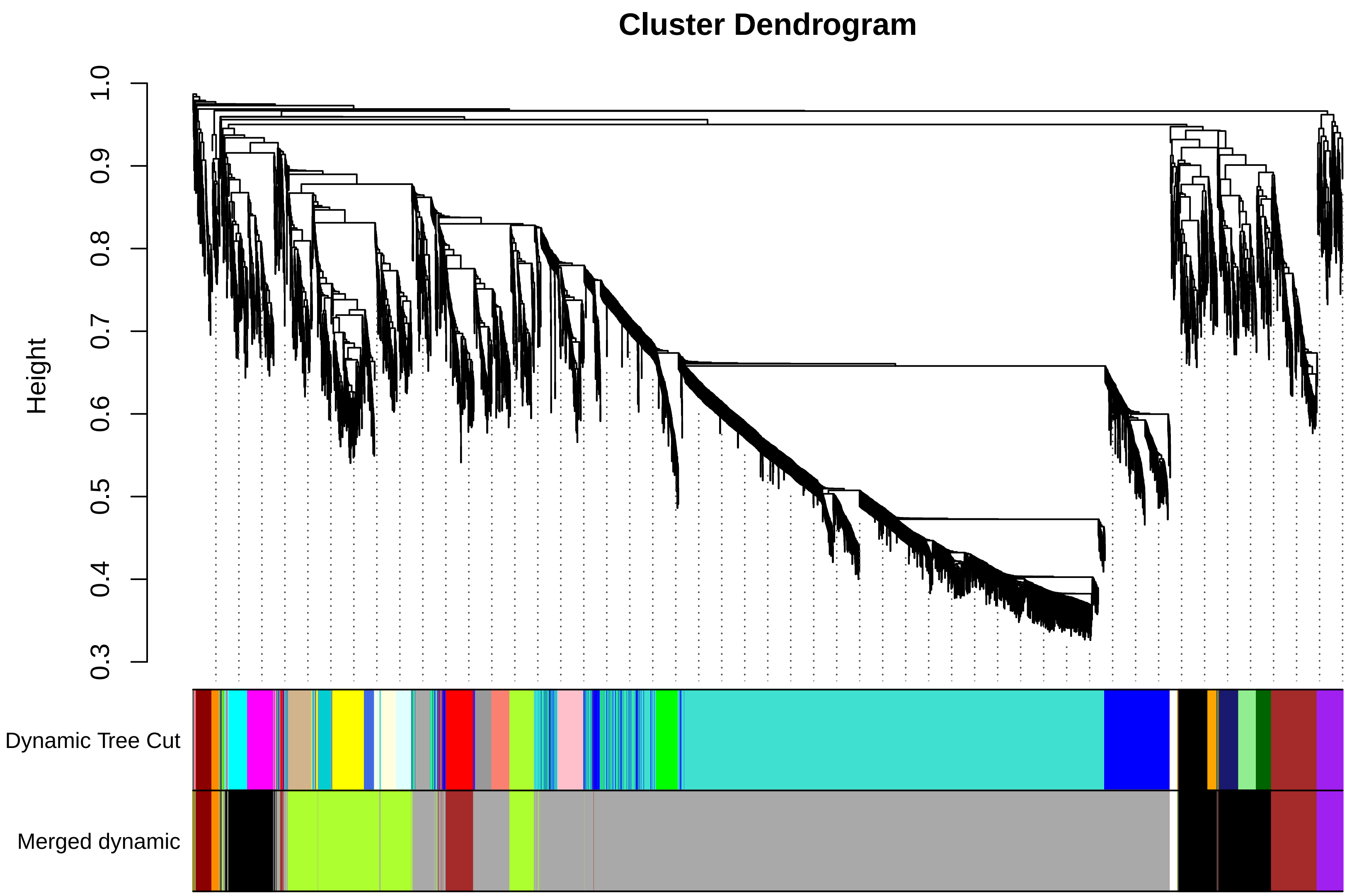
Cluster Dendrogram
Height
1.0
0.9
0.8
0.7
0.6
0.5
0.4
0.3
Dynamic Tree Cut
Merged dynamic
