# Supplementary material for: Genome-Wide Characterization of the bZIP Gene Family in Winter Jujube Highlights ZjbZIP7’s Association with Late Fruit Development
Source: Genes (Basel). 2026 Sep 30;17(10):1217. doi: 10.3390/genes17101217 (PMC13704098; doi:10.3390/genes17101217)
Supplement: Supplementary file 1 [file genes-17-01217-s001.zip › Table S1.pdf]

**Table S1.** Primer sequences used in this study.

| Primer name | Sequence (5′–3′) | Annotation |
|---|---|---|
| ZjActin-F | CTGAAGAGAACTGCCAGAAGAG | Primers for qRT-PCR |
| ZjActin-R | CTGTCCTTGGAACTGGCATAG | |
| Q-ZjbZIP7-F | GTTGGATGAATTACCGGAGG | |
| Q-ZjbZIP7-R | AGTCTCCTGCATTCTCCTTC | |
| Q-ZjbZIP9-F | ATTGGAGTGGTCGGATTAGG | |
| Q-ZjbZIP9-R | CAACTCCATAGTGTATGCCTG | |
| Q-ZjbZIP11-F | CATCAATATGTGTGGGTAGTCC | |
| Q-ZjbZIP11-R | AGACTAGGAGTACCAAGCTG | |
| Q-ZjbZIP21-F | TCGGATGCGAAAACAGAAGC | |
| Q-ZjbZIP21-R | AATTCCAGGGATTCACAAACCC | |
| Q-ZjbZIP29-F | AGGGTTGAGCACTCTACTTTG | |
| Q-ZjbZIP29-R | GAAGCTGCTGTCTAATCTCTG | |
| Q-ZjbZIP30-F | AAACAACCTTTAGCCATGGACC | |
| Q-ZjbZIP30-R | AACTGGCATCTCTATCCTCC | |
| Q-ZjbZIP31-F | ATGGCTTCCTCAAGTGGTACAT | |
| Q-ZjbZIP31-R | GGAGTTCTCAGACTCAATGG | |
| Q-ZjbZIP43-F | AGGTTAAAGGGCATTGCAGC | |
| Q-ZjbZIP43-R | TATCAGCTTGGTGAAGGAATCC | |
| AD-ZjbZIP7-F(EcoRI) | GCCATGGAGGCCAGTGAATTCATGGAAGAAGAGCTGGAATT | Primers for Y1H |
| AD-ZjbZIP7-R(BamHI) | CTCGAGCTCGATGGATCCAGAACAAGGAAACACTGTT | |
| PAbAi-proZjACO1-F(BamHI) | CTTGAATTCGAGCTCGGTACCTCTGAGATATTCATA | |
| PAbAi-proZjACO1-R(XhoI) | ATACAGAGCACATGCCTCGAGCGTTAACTCTCAGGC | |
