# Supplementary material for: Genome-Wide Characterization of the bZIP Gene Family in Winter Jujube Highlights ZjbZIP7’s Association with Late Fruit Development
Source: Genes (Basel). 2026 Sep 30;17(10):1217. doi: 10.3390/genes17101217 (PMC13704098; doi:10.3390/genes17101217)
Supplement: Supplementary file 1 [file genes-17-01217-s001.zip › Table S2.pdf]

Table S2. Physicochemical properties and predicted subcellular localization of the 52 ZjbZIP proteins identified in winter jujube. MW, molecular weight; pI, theoretical isoelectric point; GRAVY, grand average of hydropathicity.

gene name	Gene id	Chromosome	Protein Length (aa)	M W(Da)	PI	Instability Index	Aliphatic Index	Grand Average of Hydropathicity	Stability	Subcellular Localization
ZjbZIP1	LOC107417784	Chr1	264	29307.66	5.69	34.2	69.81	-0.756	D	nucleus
ZjbZIP2	LOC107408598	Chr1	229	25550.97	9.06	65.3	89.83	-0.516	G	nucleus
ZjbZIP3	LOC107405095	Chr2	427	46237.98	6.09	57.4	60.66	-0.785	B	nucleus
ZjbZIP4	LOC107422092	Chr2	143	16153.24	9.3	58.46	58.74	-1.069	F	nucleus
ZjbZIP5	LOC125421818	Chr3	323	36024.72	6.01	54.32	63.5	-0.861	B	nucleus
ZjbZIP6	LOC107416508	Chr3	196	22785.29	6.13	60.88	68.62	-0.916	G	nucleus
ZjbZIP7	LOC107416075	Chr4	307	34072.51	4.61	64.04	89.93	-0.367	C	nucleus
ZjbZIP8	LOC125423064	Chr4	235	25376.05	7.61	78.92	48.21	-0.691	L	nucleus
ZjbZIP9	LOC107420002	Chr4	470	50760.93	7.75	35.62	68.09	-0.592	E	nucleus
ZjbZIP10	LOC107420484	Chr4	157	18036.2	7.94	57.97	77.07	-0.847	L	nucleus
ZjbZIP11	LOC107406320	Chr4	349	38379.34	5.49	51.51	64.53	-0.682	H	nucleus
ZjbZIP12	LOC107422670	Chr4	325	36397.92	5.39	51.58	80.12	-0.534	E	nucleus
ZjbZIP13	LOC107422693	Chr4	411	46212.46	7.18	49.02	81.95	-0.372	A	nucleus
ZjbZIP14	LOC107421189	Chr4	167	18168.02	9.64	62.06	64.37	-1.084	C	nucleus
ZjbZIP15	LOC107421670	Chr4	313	33876.96	9.09	46.75	70.16	-0.685	E	nucleus
ZjbZIP16	LOC107403423	Chr4	339	37067.26	9.09	51.29	63.95	-0.836	B	nucleus
ZjbZIP17	LOC107403268	Chr5	367	39461.31	5.97	63.83	49.84	-0.934	F	nucleus
ZjbZIP18	LOC107430881	Chr5	222	25348.95	5.48	42.06	99.77	-0.312	A	cytosol
ZjbZIP19	LOC107423733	Chr6	427	45977.98	6.54	49.69	64	-0.775	F	nucleus
ZjbZIP20	LOC107424454	Chr6	316	35714.65	9.92	58.94	62.41	-0.642	L	nucleus
ZjbZIP21	LOC107424569	Chr6	157	18014.19	6.42	61.51	77.07	-0.748	L	nucleus
ZjbZIP22	LOC107424811	Chr6	354	37305.12	6.56	50.58	53.87	-0.84	F	nucleus
ZjbZIP23	LOC107424997	Chr6	202	23269.16	9.94	68.68	61.24	-0.804	G	nucleus
ZjbZIP24	LOC107425422	Chr6	363	41012.5	7.07	42.39	76.67	-0.487	A	nucleus
ZjbZIP25	LOC107428714	Chr7	396	44136.32	6.69	59.71	78.91	-0.6	A	nucleus
ZjbZIP26	LOC107412942	Chr7	321	35584.09	8.5	59.61	66.88	-0.807	E	nucleus
ZjbZIP27	LOC107414005	Chr7	383	41974.24	5.78	66.43	60.97	-0.826	B	peroxisome
ZjbZIP28	LOC107406060	Chr8	146	16504.75	8.11	55.56	85.55	-0.577	L	nucleus
ZjbZIP29	LOC107425940	Chr8	442	47569.88	6.43	55.03	71.11	-0.641	H	nucleus
ZjbZIP30	LOC107426725	Chr8	588	64249.72	6.35	64.45	56.62	-0.888	B	nucleus
ZjbZIP31	LOC107406434	Chr8	170	18843.93	6.3	48.54	75.24	-0.566	L	nucleus
ZjbZIP32	LOC107404439	Chr8	362	39990.94	6.05	48.39	55.28	-0.988	F	nucleus
ZjbZIP33	LOC107427086	Chr8	436	46235.87	9.85	52.33	66.93	-0.614	E	nucleus
ZjbZIP34	LOC107427517	Chr18	272	29467.89	6.21	37.2	58.53	-0.66	D	nucleus
ZjbZIP35	LOC107421564	Chr8	295	32249.12	7.79	68.34	63.83	-0.781	E	nucleus

ZjbZIP36	LOC107409642	Chr9	474	52642	8.82	55.84	64.58	-0.798	E	nucleus
ZjbZIP37	LOC107409892	Chr9	514	56968.72	7.37	57.42	69.34	-0.58	A	nucleus
ZjbZIP38	LOC107432156	Chr9	288	32464.47	9.32	48.95	76.28	-0.739	E	nucleus
ZjbZIP39	LOC107411485	Chr9	313	35335.63	9.32	48.99	72.91	-0.857	B	nucleus
ZjbZIP40	LOC107411572	Chr9	470	49854.65	6.37	59.01	54.06	-0.816	B	nucleus
ZjbZIP41	LOC107412221	Chr9	437	47195.03	6.06	55.43	58.97	-0.815	H	nucleus
ZjbZIP42	LOC107431532	Chr10	749	82145.02	7.07	50.31	71.6	-0.592	C	Endoplasmic reticulum
ZjbZIP43	LOC107403961	Chr10	467	51490.68	7.09	51.35	78.82	-0.558	A	nucleus
ZjbZIP44	LOC107434956	Chr10	513	57140.04	8.89	59.74	64.33	-0.808	F	nucleus
ZjbZIP45	LOC107432186	Chr10	438	48065.62	8.47	61.51	63.88	-0.787	E	nucleus
ZjbZIP46	LOC107405484	Chr10	513	57349.55	7.77	60.44	68.32	-0.685	A	nucleus
ZjbZIP47	LOC107432516	Chr10	343	38339.63	6.25	67.82	60.85	-0.872	B	nucleus
ZjbZIP48	LOC125419655	Chr10	171	19144.45	8.84	57.56	59.42	-0.984	F	nucleus
ZjbZIP49	LOC107418952	Chr10	127	14671.01	9.96	72.67	82.13	-0.612	L	nucleus
ZjbZIP50	LOC107433384	Chr10	202	22855.75	9.66	70.09	71.98	-0.96	C	nucleus
ZjbZIP51	LOC125418709	Chr11	207	23508.1	6.25	53.87	78.7	-0.8	G	nucleus
ZjbZIP52	LOC107429500	Chr11	442	48747.75	5.96	39.35	79.5	-0.348	A	nucleus
